# Oxidative stress in female cancers

**DOI:** 10.18632/oncotarget.25323

**Published:** 2018-05-04

**Authors:** Gloria M. Calaf, Ulises Urzua, Lara Termini, Francisco Aguayo

**Affiliations:** ^1^ Instituto de Alta Investigación (IAI), Universidad de Tarapacá, Arica, Chile; ^2^ Center for Radiological Research, Columbia University Medical Center, New York, NY, USA; ^3^ Departamento de Oncología Básico Clínica, Facultad de Medicina, Universidad de Chile, Santiago, Chile; ^4^ Instituto do Câncer do Estado de São Paulo, Centro de Investigação Translacional em Oncologia, Laboratório de Oncologia Experimental, São Paulo, SP, Brazil; ^5^ Advanced Center for Chronic Diseases (ACCDiS), Universidad de Chile, Santiago, Chile

**Keywords:** oxidative stress, cervical, breast, ovarian cancer, curcumin

## Abstract

Breast, cervical and ovarian cancers are highly prevalent in women worldwide. Environmental, hormonal and viral-related factors are especially relevant in the development of these tumors. These factors are strongly related to oxidative stress (OS) through the generation of reactive oxygen species (ROS). The OS is caused by an imbalance in the redox status of the organism and is literally defined as “an imbalance between ROS generation and its detoxification by biological system leading to impairment of damage repair by cell/tissue”. The multistep progression of cancer suggests that OS is involved in cancer initiation, promotion and progression. In this review, we described the role of OS and the interplay with environmental, host and viral factors related to breast, cervical and ovarian cancers initiation, promotion and progression. In addition, the role of the natural antioxidant compound curcumin and other compounds for breast, cervical and ovarian cancers prevention/treatment is discussed.

## INTRODUCTION

Epidemiological and experimental studies have shown that excessive production of reactive oxygen species (ROS) may lead to consequent alteration in the intracellular homeostasis and cause damage to important components of the cells when the excess of oxidants is not balanced by antioxidant defense and/or DNA repair mechanisms [1]. Oxidative stress (OS) is caused by an imbalance in the redox status of the body and is literally defined as “an imbalance between ROS generation and its detoxification by biological system leading to impairment of damage repair by cell/tissue”. ROS include superoxide anion radical (O2), singlet oxygen (1O2), hydrogen peroxide (H_2_O_2_) and the highly reactive hydroxyl radical (OH^-^). They can be generated due to naturally occurring processes that increase free radicals in the body and lead to tissue damage such as environmental stimuli and pollutant (e.g., ionizing radiation), change in atmospheric conditions (e.g., hypoxia), lifestyle stress (e.g., cigarette smoking, alcohol consumption, etc.) [2]. The chronic OS can drive to carcinogenesis by altering expression of cancer-related genes causing transformation due to mutations [1]. ROS can have multiple effects in the initiation stage of carcinogenesis mediating carcinogen activation, causing DNA damage, and interfering with the DNA damage response (DDR) [3–5]. Cancer cells exhibit higher levels of ROS compared to normal cells. Since increased antioxidant defense balances the oxidative status within the cancer cells it has been suggested that high ROS levels may prevent carcinogenesis via several mechanisms [6].

One of the most important species of free radicals is ROS produced by various metabolic pathways, including aerobic metabolism in the mitochondrial respiratory chain. It plays a critical role in the initiation and progression of various types of cancers. ROS affects different signaling pathways, including growth factors and pathways of mitosis, and controls many cellular processes, including cell proliferation, and uncontrolled growth of cells leading to carcinogenesis. Increased OS caused by ROS can reduce the antioxidant defense of the body against angiogenesis and metastasis in cancer cells that are the main processes in cancer development [7].

## BRIEF EPIDEMIOLOGICAL ASPECTS OF FEMALE CANCERS

### Cervical cancer

Cervical cancer is the third most common cancer in women and accounts for 12% of women affecting cancers in less developed countries [8]. In fact, in 2012 the World Health Organization (WHO) estimated that 528,000 women were affected by this disease in the world. In addition, cervical cancer is the most important cause of death in women in a fertility age [9]. The prevalence of this cancer is highly dependent of high-risk human papillomavirus (HR-HPV) infection. Approximately 70% of cervical cancer worldwide is etiologically related to only two HR-HPV types: HPV-16 and -18 [10].

### Breast cancer

Global cancer burden increased to 14.1 million new cases in 2012; and breast cancer is the most common cancer in women worldwide, with nearly 1.7 million new cases diagnosed in 2012 [11]. Breast cancer is the most frequent spontaneous malignancy diagnosed in women in the western world and it is a classic model of hormone-dependent malignancy. Estrogen has generally been considered beneficial, based on a variety of hormonal effects; however, in the past 15 to 20 years, epidemiological studies have increasingly pointed to an increased breast risk associated with them.

### Ovarian cancer

Ovarian cancer (OC) is the eighth most common cancer and the seventh leading cause of cancer death in women worldwide. Due to lack of efficient early screening tools and the absence of specific disease signs, over 70% of patients are diagnosed at stages III-IV when disease has typically spread to the peritoneal cavity. The average 5-years survival is 35% and 20% for stages III-IV, respectively. Reproductive behaviours including parity, use of anovulatory contraception and breastfeeding are linked to reduced risk, whereas nulliparity increases risk [12]. In Chile, OC ranks as the second cause of death by gynecological cancer and has been recently included in a government public health initiative (AUGE) to facilitate OC treatment of low-income affected patients [13].

## ORIGIN OF FEMALE CANCERS

### Human papillomavirus in cervical cancer

Human papillomavirus (HPV) belongs to papillomaviridae family and its genome is an 8,000 base pairs double-stranded DNA which contains two coding regions (E and L) and one non-coding region named long control region (LCR) with regulatory functions. The early (E1, E2, E4, E5, E6 and E7) and late (L1 and L2) HPV proteins are expressed from polycistronic and polyadenylated mRNAs processed by alternative splicing [14]. There are five HPV genders: alpha, beta, gamma, mu and nu papillomavirus although the causal agents of genital warts are alpha-papillomaviruses with mucosal and cutaneous tropism [15]. HPV is able to infect susceptible cells located in the basal lamina that express the receptors involved in HPV entry: heparan sulphate proteoglycans and integrin family proteins [16]. The virus enters the cells by an endocytosis-mediated mechanism and later replicates into the nucleus, using the host replication and repair machinery. The viral gene expression is finely regulated by the Long Control Region (LCR), located upstream of the early promoter. This region contains an enhancer region where cellular transcription factors bind to cognate binding sites and regulate the activity of the early promoter which is located next to the E6 start codon (nucleotide 97 in HPV-16 and 105 in HPV-18). A consensus TATA box located upstream of the transcription initiation site recruits the TFIID transcription factors. Upstream of the initiation start site, E2 binds to its cognate sites located in the early promoter (known as E2 binding sites or E2BS), repressing its activity. The transcription factors activator protein 1 (AP-1) and specificity protein 1 (SP-1) are the most important cellular regulators of HPV gene expression [17]. Mutations in AP-1 binding sites into the LCR completely shut down HPV early promoter activity in cell lines, demonstrating the importance of AP-1 for HPV replication and gene expression [17]. The HPV early promoter is active in diverse epithelial cell lines, as previously reported [18]. The HPV early and late proteins are expressed from polycistronic and polyadenylated transcripts processed by alternative splicing and no information is available on how the mechanism of translation initiation occurs for each viral gene. However, it was reported that apparently E7 is translated using a non-canonical mechanism through leaky scanning [19]. The most prevalent HR-HPV types in cervical carcinomas are HPV-16, 18, 31, 33, 35, 45, 52 and 58 among others [20]. It is considered that almost 100% of cervical carcinomas worldwide are HR-HPV positive [10].

Cervical cancer initiation is strongly related to HR-HPV infection. The HR-HPV is able to modulate signaling pathways associated to cell cycle control, apoptosis, DNA damage response (DDR) and others although the HR-HPV oncogenic potential is strongly related to E6 and E7 oncoprotein expression. A very important event in HR-HPV-mediated oncogenicity is the overexpression of E6 and E7 oncoproteins, generally by viral integration into the host genome [21]. As a consequence, the tumor cells become addicted to E6 and E7 overexpression [22]. Currently, it is accepted that HR-HPV E6 and E7 oncoproteins are able to immortalize cells, however for complete tumor transformation, additional alterations are strongly required. Genetic, host or environmental cofactors are related to the ability of HR-HPV to persist into the tissue with an increased risk to promote cancer development. Genetic polymorphisms, immune status and environmental carcinogens, among others, may indicate an increased risk of HR-HPV-mediated carcinogenesis [23, 24].

Integration of HR-HPV genome into the host chromosome is a frequent event in cervical carcinogenesis. It is assumed that in early lesions as cervical intraepithelial neoplasia (CIN1), the HR-HPV is frequently found episomal and in late lesions (CIN2-3) such virus is integrated. HR-HPV integration frequently disrupts the E2 region, resulting in the subsequent loss of gene function. However, integration may also occur in other regions within the viral genome such as L1 or E1 [25]. In addition, it has been reported that host genome integration sites are randomly distributed [26]; even though some preferential hot spots have been detected, for instance in the gene encoding for FHIT, DLG2, MYC and SEMA3D among others [27]. The E6 oncoprotein is a 160 amino acids protein with two zinc-finger domains [28] and the E7 oncoprotein is a 98 amino acids protein that displays three functional domains. The oncogenic mechanism of HR-HPV E6 and E7 oncoproteins is related to the ability of the virus to bind and induce proteosomal and ubiquitin-dependent p53 and pRb degradation with the subsequent loss of apoptosis and cell cycle control, respectively [29]. In addition, HR-HPV E6 and E7 oncoproteins interact with a plethora of other proteins in epithelial cells, causing additional molecular alterations [30]. The other E6 partners are: transcriptional co-activators, PDZ domain proteins, tumor suppressor proteins and inducers of apoptosis and factors of DNA replication and repair [31]. It has been reported that E6 oncoprotein induces an increased expression and activity of telomerase, which is assumed to be responsible for E6-mediated immortalization [32, 33]. On the other hand, the HR-HPV E7 binding to pRb family proteins induces E2F releasing and overexpression of p16^INK4a^ [34]. It has been found that pRb knockout mice do not recapitulate the E7-mediated transformation properties, suggesting other E7 interactions [35]. In fact, E7 targets p107 and pRb2/p130 pocket family of proteins for degradation [36, 37]. Both low-risk (LR) and HR-α-HPV E7 proteins are able to induce the degradation of pRb2/p130 protein [37]. Other E7 protein partners include cyclins, p21 and p27, which causes cell immortalization [35].

### Hormone and environmental factors in breast cancer

Over the past 15–20 years, epidemiological studies have pointed to an increased breast cancer risk associated with prolonged exposure to female hormones. Major advances in our understanding of the etiology of cancer and its causative mechanisms have been done in the past decades. There are substantial evidences that breast cancer risk is associated with prolonged exposure to female hormones, since onset of menarche, late menopause, and hormone replacement therapy are associated with greater cancer incidence and since about one third of breast cancers is responsive to endocrine therapies [38].

Breast cancer may have its genesis and cell growth influenced by hormonal factors; however, the potential carcinogenic activity of estrogen-containing medications in humans has not been recognized for many years. Authors [39, 40] have shown that estrogen administration, a risk factor for humans, increases with continuous doses of estrogen and with the length of treatment. Slightly elevated levels of circulating estrogen are also a risk factor for breast cancer. Several studies have demonstrated strong relationships between endogenous estrogen levels and breast cancer risk [41–43]. This role of endogenous estrogen in human breast carcinogenesis is supported by risk factors of breast cancer such as high serum or urine estrogen levels.

Estrogen is associated with carcinogenesis in humans and animals [44–49], and the exact effect of estrogen in breast cancer remains unclear at this time. Estrogen has been implicated in a variety of cancers. Since that time, many more reports on tumor induction by estrogen have been published, and many rodent tumor models have been reviewed [50]. The evidence for the carcinogenic activity of estrogen in animals has been reported by those groups. This conclusion was based on numerous experiments related to the administration to rodents of oral or subcutaneous estrogen, which resulted in an increased incidence of mammary tumors [51, 52]. Latest data from International Agency for Research on Cancer (IARC) shows that breast cancer is the leading cancer site in women and is the leading cause of death among female cancers. Environmental substances seem to be involved in the etiology of breast cancers, the most frequent malignancy diagnosed in women and is a classical model of hormone-dependent malignancy [38]. On the other hand, environmental chemicals such as parathion and malathion, organophosphorous pesticides used to control a wide range of sucking and chewing pests of field crops, are involved in the etiology of breast cancers [53]. The use of these organophosphorous insecticides in agriculture and in urban settings has increased significantly. Many studies have found an association between human cancer and exposure to agricultural pesticides as demonstrated by IARC [54, 55]. Our study demonstrated that parathion and malathion induced tumor formation in a specific target organ, the mammary gland. This was the first report that organophosphorous pesticides induced changes in mammary gland associated with carcinogenesis. Our results showed that initiation occurs primarily in the epithelium of terminal end buds while they are developing into alveolar buds because these structures were affected by pesticides. This is an important finding because such structures are considered equivalent to the terminal ductal lobular unit described in the human breast. Treatment with these substances induced significant changes at a cellular level [56–58].

ROS-mediated OS is known to play a role in breast cancer pathogenesis via genetic and epigenetic modifications, resulting in uncontrolled cell proliferation. Induction of ROS and OS as a consequence of impaired balance between pro-oxidants and antioxidants are suggested to be involved in induction and progression of breast cancer [44, 45, 47–49], and the exact effect of estrogen in breast cancer remains unclear at this time. Estrogen has been implicated in a variety of cancers. Since that time, many more reports on tumor induction by estrogen have been published, and many rodent tumor models have been reviewed [50]. The role of OS in the etiology of this cancer is supported by multiple lines of evidence [59]. Although ROS are generally thought of as damaging to cells due to their ability to induce OS at high concentrations, low levels of ROS are actually essential to normal cell function [60]. It is known that levels of ROS are often up regulated in cancer cells, and their role in promoting certain signaling cascades is likely one reason that this adaptation is advantageous [61]. It has been evident that free radicals are contributory agents either to initiate or prolong such effects. This is in part due to the fact that ROS can act as second messengers in signaling cascades that are vital for cellular responses to external stimuli [60]. Exposure to alpha particles has been associated with ROS formation [62]. Generation of hydrogen peroxide by oxidative metabolism of estrogens has been identified in tissues of breast cancer versus controls [63].

To gain insight into the effects of curcumin on OS an established *in vitro* experimental breast cancer model (Alpha model) was used [64]. This model was developed with the immortalized human breast epithelial cell line, MCF-10F [65] that was exposed to low doses of high LET (linear energy transfer) α particles (150 keV/μm) of radiation, values comparable to α particles emitted by radon progeny, and subsequently cultured in presence or absence of 17β-estradiol (estrogen). Such model gave us the opportunity to study alterations induced by OS. This model consisted of human breast epithelial cells in different stages of transformation: i) a control cell line, MCF-10F, ii) an estrogen-treated cell line, named Estrogen, iii) a malignant cell line, named Alpha3 and iv) a malignant and tumorigenic, cell line named Alpha5 and Tumor2 derived from cells originated from a tumor after injection of Alpha5 cell line injected in the nude mice. Our work indicated that curcumin inhibited cell proliferation, invasion, angiogenesis and metastasis of different cancers through interaction with multiple cell signaling proteins [66]. Various molecular targets modulated by this agent include transcription factors, growth factors and their receptors, cytokines, enzymes, and genes regulating cell proliferation and apoptosis [67–71].

### Reproductive factors in ovarian cancer

Supported by epidemiological studies that associate pregnancy and oral contraceptive use to reduced ovarian cancer (OC) risk, repetitive ovulation has been implicated in OC initiation based on damage incurred to the ovarian surface epithelium (OSE) upon continuous cycles of tear and repair of this cell layer during fertile life of women [12]. Accordingly, OSE cells have been widely studied as candidates of OC origin [72]. The OSE has a mixed or uncommitted phenotype that expresses epithelial (cytokeratins 7, 8, 18 and 19), mesothelial (MUC1 and HSD17B) and mesenchymal (N-cadherin, vimentin and α-actin) markers. In addition, a number of hormone receptors including those of estrogen, progesterone, gonadotropins, androgens and activin/inhibin have been detected in the OSE [72, 73].

The ovulation that temporarily disrupts the OSE resembles a pro-inflammatory process [74]. The surge of luteinizing hormone (LH) occurring by mid fertile cycle induce an increase of ROS levels thus triggering a sequence of signaling, biochemical and cell remodeling events in both ovarian follicles and the OSE [75]. Indeed, ovulation can be impaired by ROS scavengers placed in the ovarian bursa of mice [76]. At the site of follicular rupture, OSE cells undergo apoptosis and adjacent cells become exposed to ROS and inflammatory signals [77]. Follicular fluid has been demonstrated to contain high ROS levels inducing to oxidative stress and DNA damage in epithelial cells of fimbriae compared to the isthmus segment of the fallopian tube [78]. Increased expression of genes associated to inflammation and to OC precursor lesions occurs in human and bovine oviductal cells in response to follicular fluid exposure [79, 80].

Early culture passages of primary mouse OSE cells experience a subtle pulse of oxidative stress-related gene expression with subsequent mitotic alterations leading to aneuploidies that drive their malignant transformation [81]. Both OSE cells in ovarian organoids and 2-D cultured MOSE cells show AKT1-mediated DNA damage and increased proliferation upon exposure to hydrogen peroxide [82]. In addition, OSE transformation depends on an interaction with the underlying stroma as suggested by enhanced proliferation of c-myc expressing OSE cells when placed in contact with OS-induced senescent human fibroblasts [83].

Immersed in the ovarian stroma, female germ-cells (oocytes) within follicles are increasingly exposed to OS -presumably resulting from steroidogenic p450 enzyme activity- as follicles progress through sequential maturation stages [84]. This key reproductive process, folliculogenesis, can be affected by a wide variety of endogenous and exogenous agents that modulate ovarian ROS levels and may in turn affect the oocyte reserve [85]. As a part of the natural history of the mammalian ovary, less than 1% of the total oocyte reserve is ovulated during fertile life of woman. The remaining oocytes contained in ovarian follicles undergo *atresia*, a cyclic degenerative process initiated by apoptosis of granulosa cells [86] in which JNK/FoxO1 pathway-mediated oxidative stress has been recently implicated in mouse [87].

Therefore, the ovarian homeostasis depends on a delicate redox balance between ROS levels essential for reproductive functions and antioxidant enzymes plus steady levels of reduced glutathione (GSH), the major endogenous antioxidant metabolite, that combined counteract the deleterious effects of excess ROS [88]. The transcription factor NRF2 stimulates the expression of detoxifying and antioxidant genes thus preserving the homeostasis of the primordial follicle pool. Indeed, null *Nrf2* mice exposed to the toxicant benzo-a-pyrene (BaP) display an accelerated ovarian follicle depletion by middle-age [89]. The rate-limiting step of GSH synthesis is catalyzed by the heterodimeric enzyme glutamate cysteine ligase, composed of a modifier (*Gclm*) and a catalytic (*Gclc*) subunit. When null *Gclm* mice are exposed prenatally to BaP, early apoptosis of growing follicles and cytokeratin-positive ovarian tumors are observed later in life [89].

Interestingly, atresia can be prevented by follicle stimulating hormone (FSH) through inhibition of antral follicle apoptosis by decreasing ROS and recovering GSH levels [90]. Modulation of autophagy, a non-apoptotic cell death mechanism, would be also important in atresia of mouse granulosa cells (GC). HIF-1α signaling promoted GC autophagy upon exogenous FSH exposure [91], whereas under OS, FSH would counteract autophagy of GCs via acetylated-FOXO1 [92]. Thus, the effect of FSH on ROS levels might appear paradoxical in two aspects: i) gonadotropins stimulate follicular steroidogenesis and thereby, OS [84]; and ii) increased levels of gonadotropins at menopause have been implicated in ovarian carcinogenesis [93]. Intriguingly, in post-menopausal ovaries the OSE, its derived inclusion cysts and the ovarian stroma express aromatase (*Cyp19a1*) and gonadotropin receptors [94]. Since post-menopause is a state approaching follicle exhaustion with concomitant oxidative damage to the ovary [95], the occurrence of this residual steroidogenic capacity and gonadotropin responsiveness deserves further research regarding their roles in the redox status of the post-reproductive ovary and its contribution to OC initiation and development.

## PROGRESSION OF FEMALE CANCERS

### Cervical cancer

Although HR-α-HPV infection and overexpression of viral oncoproteins are strongly related to cancer initiation, viral-related factors are associated to both promotion and progression. However, it is necessary to appoint that overexpression of HR-α-HPV oncoproteins, although always necessary, is not a sufficient condition for cervical cancer progression. In cell models, E6 and E7 oncoproteins efficiently immortalize cervical keratinocytes, although are unable to promote full cell transformation. Actually, for complete cell transformation, other factors are strongly required. Tobacco smoking, oral contraceptives use, immunosuppression, microbiome in the vagina, are some of the suggested cofactors potentially involved in HR-α-HPV mediated cervical cancer promotion and progression. In addition, OS is an under-explored factor, common to the suggested cofactors, eventually having a role in cervical cancer, working either synergistically or independently to HR-α-HPV infection. It has been previously reported the existence of higher levels of oxidative DNA damage in HPV-positive cervical lesions compared to control samples. The levels of damage were particularly high in high-grade squamous intraepithelial lesions, which are closely associated with high-risk HPV types [96, 97].

Chronic inflammation associated to HR-HPV-persistent infection may lead to the production and release of ROS. In HR-HPV-infected cells, OS is mainly induced by the expression of viral oncoproteins. Besides, during HPV infections ROS can be also produced by activated neutrophils and macrophages associated to the local immune response. Altogether, alterations in infected cell metabolism and the non-effective chronic inflammatory response may contribute to cell transformation [98–101].

HR-HPV integration or LCR methylation leads to the up-regulation of E6/E7 proteins increasing the number of chromosomal rearrangements and DNA lesions. Besides, genetic instability caused by the oxidative stress triggered by cervical carcinogenesis co-factors such as tobacco smoking, oral contraceptives, infections by other agents associated with genital disease, could also increase the risk of HR-HPV integration. Moreover, p53 and pRb degradation mediated by E6 and E7, respectively, promotes alterations in cell cycle and metabolism regulation. Finally, epigenetic and genetic modifications in host and viral genomes can further contribute to the deregulated expression of host genes associated to cell proliferation control and, ultimately, to cervical carcinogenesis [102, 103].

HR-HPV is able to modulate several antioxidant defenses counteracting the effect of increased ROS levels on infected cells viability allowing them to proliferate even in the presence of DNA lesions. For instance, HPV oncoproteins allow infected cells to survive in an increasing oxidant environment by down-regulating the oxidation of antiapoptotic and detoxifying enzymes together with redox-sensitive transcription factors. Besides, E6 and E7 modulate cellular microRNAs that regulate genes associated with antioxidant response, suppression of OS induced apoptosis and regulation of antioxidant enzymes and compounds [102]. In this way, the expression and/or activity of several antioxidant proteins including catalase, peroxiredoxins, quinone oxidoreductase-1 and superoxide dismutase (SOD) family proteins are deregulated in pre-neoplastic tissues associated to HR-HPV infections. For instance, the expression of superoxide dismutase-2 (SOD2), considered one of the most important antioxidant enzymes in the regulation of cellular redox state in normal and tumorigenic cells, is up-regulated in several tumors, including penile and cervical carcinomas, when compared to normal tissues [104–107]. Moreover, HR-HPV oncoproteins expression is associated to elevated levels of detoxifying enzymes, such GSTs and GSH, conferring to the HPV host cell an improved oxidative damage detoxifying system. Finally, resistance of HPV infected cells to programmed cell death induced by oxidant conditions is achieved by up-regulation of some apoptosis inhibitors such as surviving and IAPs (cellular inhibitor of apoptosis family).

Increasing ROS production while upregulating antioxidant enzymes constitutes a paradoxical behavior that cervical cancer shares with other malignant tumor types. While the induction of an oxidant milieu may promote tumor progression the induction of antioxidant systems may allow tumor cells to adapt to the harsh conditions of the tumor microenvironment. Taking together, these observations suggest that OS, chronic inflammation, epigenetic alterations and expression of HR-HPV oncoproteins can act in a synergistic way during cervical cancer progression.

NF-kB signaling pathway is strongly related with cervical cancer even though the evidence about the activation or suppression of NF-κB by E6 and E7 oncoproteins in cervical cancer is conflicting [108]. It has been suggested that pirin, a product of the PIR gene, is an oncogene that act as an OS sensor involved in NF-κB activation, through the BCL3 binding. Pirin is involved in epithelial mesenchyme transition (EMT) and metastasis [109], since in HeLa cells, this protein is able to suppress E-cadherin expression and is involved in the regulation of EMT markers. Alterations in this gene have been observed in various tumors and under conditions of OS, especially in tobacco smoke associated cancers [110, 111]. Previously, we observed by using cDNA microarrays that oral cells ectopically expressing HPV-16 E6 and E7 oncoproteins showed increased PIR levels in comparison with those cells transfected with an empty vector. In addition, we found that PIR transcripts are overexpressed in HR-HPV positive cervical cancer cells in a viral load-dependent manner [112]. In fact, Hela cells that harbor integrated HPV-18 genomes are currently used as models for studying PIR function [109, 111, 113]. Even though, it is now clear that HR-HPV E6/E7 can modulate NF-κB, the findings about pirin up-regulation in cervical cells suggest a new mechanism for HR-HPV mediated up-regulation of NF-kB activity. Understanding the relationship between HPV and NF-κB activation is crucial, and previous findings suggest the existence of a new mechanism mediated by PIR for HR-α-HPV-dependent NF-κB activation [112]. Particularly, NF-κB signaling pathway is a known mediator in stress response related to OS, inflammation and response to some viral infections. However, the role of HR-α-HPV in NF-kB activation pathway is not clear. In fact, it has been difficult to compare among studies because different HPV genes or cellular models are frequently used to analyze the activation of this signaling pathway [108, 114].

### Breast cancer

Breast cancer progression follows a complex multistep process that depends on various exogenous (diet, breast irradiation) and endogenous (age, hormonal imbalances, proliferative lesions, and family history of breast cancer) factors [115]. Compared with normal cells, cancer cells usually demonstrate aberrations in oxidative metabolism and signaling pathways, characterized by increased levels of reactive species. Reactive species overproduction could induce tumorigenesis and progression possibly by modulating the expression, degradation, post-translational modifications [116]. Among the oncogenes, c-Ha-Ras and Rho-A (Ras homologous A from the Ras super family) have been shown to promote both cell proliferation and invasion indicating their importance in malignant transformation [117–123]. A critical step in the stimulation of cell surface receptors by their ligand involves the accumulation of Ras proteins in their active GTP-bound state. To reach their active GTP bound state, Ras proteins must first release bound GDP mediated by a guanine nucleotide releasing factor (GRF). Members of Rho family proteins sometimes act downstream of Ras. To analyze the Ras active GTP bound state, the RasGRF1 protein expression was assessed. Results indicated that curcumin decreased RasGRF1 protein expression in the control MCF-10F, Apha5 and Tumor2 cell lines by fluorescence staining intensities. RasGRF1 protein expression decreased in Alpha3, Apha5 and Tumor2 cell lines. The Rho GTPases, identified as regulators of cytoskeletal reorganization in addition to their effects on cell growth they have been shown to be over-expressed in human tumors [117–119]. Rho-A is a small GTPase protein known to regulate the actin cytoskeleton in the formation of stress fibers and it is generally distributed in the nuclei of cancer cells [124]. Rho-A protein expression was decreased by curcumin (15 μM) in malignant and tumorigenic Alpha5 and Tumor2 cell lines in comparison to the control cell line [66].

Cancer chemoprevention is a new challenging issue in the management of cancer. The free radical-scavenging vitamins, such as all-trans-retinol, have been shown to protect against cancer development in animal models, and may be chemopreventive in humans. Many other vitamins such as vitamin C, E, and micronutrient such as selenium may also be chemopreventive [125–127]. Retinoic acid, a natural metabolite of circulating vitamin A (retinol) and an irreversible oxidation product of retinol, is essential in maintaining the normal pathway of differentiation in epithelial tissue. Retinoic acid and a number of its analogs, both natural and synthetic (retinoids), have been shown to be effective in the prevention of a variety of cancers in experimental animals, and in reversing preneoplastic lesions in humans [127].

The identification of genes involved in the prevention of breast cancer and the mechanisms by which genes participate in radiation- and estrogen-induced carcinogenesis are of critical importance. *In vitro* models are vital in understanding the events that drive a normal cell to cancer. Identification of factors involved in breast carcinogenesis has been facilitated by studies using breast cancer cell lines representative of different tumor phenotypes. Active oxygen species and other free radicals have long been known to be mutagenic. Furthermore, there is evidence that oxyradicals can modulate phenotypic and genotypic changes that ultimately lead to neoplasia [128]. In the present study, retinol treatment was found to decrease free radical production suggesting that oxyradicals play a role in carcinogenesis. Each of the cell types examined had significantly elevated H_2_O_2_ production levels compared to MCF-10F control cells. Retinol (1 μl/ml) significantly decreased H_2_O_2_ production in all cell types examined. Retinol significantly decreased invasive capabilities of cells across matrigel coated invasion chambers and significantly reduced PCNA, Fra-1, mutant p53 and increased Rb protein expression levels in comparison to non-retinol-treated ones when assayed using immunofluorescent staining coupled with confocal microscopy. The reduced H_2_O_2_ production, decrease in cell invasive capabilities and alterations in protein expression levels suggest that retinol can be used as a chemopreventive agent in human breast cancer.

### Ovarian cancer follows an atypical tumor progression mode

Epithelial OC, which accounts for nearly 90% of OC cases, can be classified as serous, mucinous, endometroid and clear-cell carcinoma. These variants are associated to Mullerian-type metaplasia, a differentiation process of OSE cells leading to formation of inclusion cysts, invaginations and epithelial crypts frequently observed in postmenopausal ovaries [129]. Notably, the ovarian follicle depletion -which is the underlying cause of menopause- is concomitant with an age-dependent impairment of the ovarian antioxidant defense [95, 130]. As OSE-derived inclusion cysts predominantly express epithelial markers when compared to the mixed phenotype of the OSE, an atypical mesenchymal-epithelial transition has been proposed for early stages of OC progression [131]. In fact, the expression of the epithelial cell marker E-cadherin is high in early ovarian carcinomas and decreases as OC cells progress to advanced stages, thus partially recovering mesenchymal features [132]. Furthermore, transcriptional profiling data from our laboratory on premalignant mouse OSE cells in culture revealed mitotic anomalies paralleled by overexpression of epithelial genes such as cytokeratins 8 and 18 along with extracellular matrix genes including fibronectin, integrin-beta 1 and the matrix metallopeptidase 2 (MMP-2) [81]. MMP-2 and MMP-9 are predominantly expressed in pre-neoplastic ovarian lesions than in advanced carcinomas [133], a finding correlated to loss of epithelial basement membrane in early lesions prior to tumor invasion and metastasis [134].

Any of the epithelial OC histotypes mentioned above may clinically behave as benign, malignant or borderline. Two broad categories have been proposed for OC development, namely type-I and type-II, reflecting that OC does not display a typical continuous progression model as described in other cancers [135]. Functional p53 gene is usually present in borderline and serous type-I tumors that grow slowly within the peritoneum becoming lethal after 2 decades of evolution. In contrast, the most common serous type-II tumors arise as rapidly-disseminating, aggressive neoplasms displaying p53 mutations as a result of *de novo* carcinogenesis [136]. Recent evidence suggests that these type-II tumors, also referred as high grade serous carcinomas (HGSC), might originate from the fallopian tube epithelium by recurrent exposure to ROS contained in the follicular fluid [78–80]. The fallopian origin of OC gained acceptance after the discovery of *p53 signatures*, normal appearing tubal mucosa containing p53 mutations, which can be regarded as latent precursor lesions preceding the formation of secretory cell outgrowths (SCOUTs) and serous tubal *in-situ* carcinoma (STIC) in women carrying BRCA mutations [137]. Then, STIC cells could exfoliate and spread to the ovary by an initial contact with the OSE [138] particularly at the time of ovulation, when an inflammatory and oxidative stress milieu is taking place [74, 76]. Whether the OSE or the fimbria is the definitive OC origin is still a matter of debate since a mixed origin cannot be ruled out with current available data and models. Finally, as observed in other malignancies, chemoresistance in advanced OC could be mediated by OS-related mechanisms [139, 140].

## CURCUMIN AS AN ANTIOXIDANT IN FEMALE CANCER TREATMENT

There is a growing awareness that OS plays a role in malignant diseases. OS is one of the important pathogenic factors of cancer development. Potential OS modulators as anticancer strategies has led to conclude that reduced antioxidants in the diet can induce specific diseases in the different organs of the body. Furthermore, there is abundant evidence that dietary and other naturally occurring antioxidants can be used to prevent or reduce such disease. However, the knowledge on fundamental biology of free radicals, especially on their molecular and cellular effects is needed for cancers. The major reason for that includes the presence of polyphenols in our diet contributing to prevention of several diseases. In addition, potent antioxidant properties of polyphenols reduce OS-associated with some diseases, including cancer. Among the polyphenols, curcumin (1, 7-bis (4-hydroxy-3-methoxyphenyl)-1,6-heptadiene-3,5-dione; diferuloylmethane) has been described as promising anticancer compounds; however, the mode of action is still unclear [141].

Phytochemicals could provide leads for the development of alternative therapeutic agents due to their antioxidant activity, as well as their ability to induce apoptosis in cancer cells [142]. Among the antioxidants, curcumin is a well-known major dietary natural yellow pigment derived from the rhizome of the herb *Curcuma longa* (Zingiberaceae). It is also named turmeric and is a perennial herb belonging to the ginger family, native to India and Southeast Asia. It measures up to 1 m high with a short stem and tufted leaves. Curcumin is present in extracts of the plant and Curcuminoids are responsible for the yellow color of turmeric and curry powder. It is a pigment of turmeric that is used for imparting color and flavor to foods. It has been shown to be a potent anti-inflammatory, antioxidant anti-carcinogenic and chemopreventive agent [143].

### Curcumin as an antioxidant in cervical cancer treatment

It has been previously reported that curcumin is able to decrease the expression of HR-α-HPV E6 and E7 oncoproteins in cervical cancer cells restoring the activity of p53 and pRb tumor suppressor proteins, respectively, suggesting the possibility that curcumin is useful for cervical cancer treatment [144]. In this respect, it was reported that curcumin induces endoplasmic reticulum stress and apoptosis through selective generation of ROS in cervical cancer cells but not in normal epithelial cells which suggest that curcumin is an effective therapeutic alternative without adverse effects in normal cells (Kim, B *et al.* 2016). The mechanisms by which curcumin may be useful for cervical cancer treatment is unclear. However, it was suggested that curcumin-mediated cytotoxicity occurs via induction of DNA damage and chromatin condensation in HeLa cells [145]. Moreover, it was reported that curcumin at low concentrations is not able to promote genotoxic damage in HeLa cells although an increase of apoptosis and superoxide levels was found [146]. Early studies by Rosl *et al.* (1997) demonstrated that HPV-16 human keratinocytes treated with the antioxidant pyrrolidine-dithiocarbamate (PDTC) altered the AP-1 heterodimer composition resulting in suppression of viral early genes expression. It would be plausible the notion that curcumin functions in a similar way [147].

However, the low aqueous solubility, poor absorption and bioavailability of curcumin has limited the potential usefulness of this compound for cancer treatment [148]. Thus, nanotechnology has emerged as an efficient alternative for curcumin incorporation into the tumor cells. In fact, new formulations with nanocarriers for co-delivery of curcumin with anti-neoplastic drugs have been tested using *in vitro* settings of cervical cancer cells [149]. In addition, formulations including other compounds such as epicatechin gallate, resveratrol and curcumin (TriCurin) have been analyzed with results showing a synergism *in vitro* and *in vivo* [150, 151].

### Curcumin as an antioxidant in breast cancer treatment

Under OS conditions, superoxide anions are produced and converted to hydrogen peroxide through a specific antioxidant system, and then to water to complete the detoxification pathway [152]. Free radicals caused by molecular reactions create compounds that can be used as indicators of cancer. Thus, oxidative damage can be measured by agents that can recognize cell damage and specific biomarkers can recognize oxidative damage [153]. It has been reported that MnSOD2 expression is up regulated in response to OS in various types of cells and tissues by toxic stimuli and treatments, such as ionizing radiation and ultraviolet light [154]. However, there is a multitude of contradictory data in human breast cancer patients. It has been reported that during progression of cancer, low levels of SOD2 lead to increased ROS that causes accumulation of mutations, thus SOD2 is increased in later stages to eliminate ROS and promote carcinogenesis. Abnormal levels of MnSOD in cancer have been documented to play a critical role in the survival of cells. It is a nuclear encoded mitochondrial antioxidant enzyme that catalyzes the conversion of superoxide radicals into molecular oxygen and hydrogen peroxide, which further reduced into water by peroxide metabolizing enzyme systems, as catalase, an endogenous antioxidant that neutralizes hydrogen peroxide by converting it into water and oxygen. An observed excess of hydrogen peroxide can be an indication of the imbalance in redox control that causes oxidative cell damage *in vitro*. Thus, hydrogen peroxide can be useful to recognize OS in the cells. Hydrogen peroxide levels were measured by the Amplex™ Red Hydrogen Peroxide Assay kit. It was interesting to find that curcumin decreased the formation of hydrogen peroxide in the cells when compared with their non-treated counterparts [66, 155]. As MnSOD the catalase are the principal defense. Catalase is a peroxisome specific marker protein that belongs to the catalase family. It is an important regulator of OS and protector of the cells from hydrogen peroxide [156]. Catalase protein expression was decreased in Alpha5 cell lines when treated with curcumin in comparison to their counterpart and Tumor2 cell line did not show any expression, indicating the low defense against OS of cells present in tumors. Our results showed that curcumin decreased SOD-2 protein expression in the control MCF-10F, Alpha3, and Tumor2 cell lines; however, MnSOD was slightly increased in the Alpha5 cell line [157].

Active oxygen produced under stress is a detrimental factor that causes lipid peroxidation [158] and the best biomarkers of this peroxidation are the Isoprostanes such as the 8-isoprostaglandinF2α (8-iso-PGF2α), malondialdehyde that show lipid damage [159–161] and it is an end product that provides a useful tool to monitor OS in human organisms in biological tissues or fluids. Isoprostanes are a novel class of prostaglandin-like compounds produced upon peroxidation of lipoproteins and may play a causative role in carcinogenesis. Our results indicated that malignant breast tissues had significantly greater 8-iso-PGF2α (pg/ml) than the normal counterpart from the same patients [155].

Aberrant COX-2 expression has been detected in many human malignancies including breast cancer. The inhibition of COX-2 enzymatic activity by specific inhibitors is an effective tool for controlling cancer progression. The suppression of prostaglandin synthesis through selective inhibition of COX-2 has been suggested as a strategy to develop chemopreventive substances. Curcumin inhibits COX-2 enzyme activity by preventing the conversion of arachidonic acid to prostaglandin during prostaglandin synthesis. The downstream product of COX-2 enzymatic activity is prostaglandin E2 (PGE2), which is an important stimulus for cell signaling pathway induction, including that of NF-κB that influences cell proliferation. In normal cells, COX-2 gene is highly inducible by signals that activate the NF-κB pathway. In contrast, many types of cancer cells possess high basal levels of COX-2, due to permanent activation of NF-κB followed by increased COX-2 gene expression [162]. We found that curcumin- decreased COX-2 protein expression in the pre-tumorigenic Alpha5 cell lines and had no effect on tumor [66, 155]. Other authors reported that curcumin decreased viability and promoted apoptosis of MCF-7 breast cancer cells by inducing caspase 3/9 activities. Moreover, curcumin downregulated miR-21 by upregulating PTEN/Akt signaling pathway [163]. Recently, by RNA sequencing and bioinformatics approaches, it was demonstrated that curcumin therapeutic potential involves the regulation of some genes such as SERPINE1, MAP3K1, GSTO2, VIM, SAPARC, and FGF2 [164].

### Curcumin in follicular homeostasis and its potential in OC therapy

The consumption of plant-derived polyphenolic compounds is associated to low risk of chronic diseases including several types of cancer. A large body of research has demonstrated that the ROS-scavenging properties of these compounds, underlie their health beneficial effects. Found in turmeric and related plants, the polyphenol curcumin (diferuloil-methane) shows anti-inflammatory, antitumoral and hypoglycemic properties by acting on multiple molecular targets in a wide range of normal and pathological cell types [165]. Curcumin inhibited cell proliferation and viability while promoted apoptosis and secretion of progesterone and testosterone in primary cultures of porcine ovarian granulosa cells [166]. When these cells were exposed to the mycotoxin zearalenone, all cellular indicators of OS were reduced upon cell pre-treatment with curcumin [167]. Further, in a model of arsenic-induced oxidative damage in mouse ovaries, curcumin reduced ROS and malondialdehyde levels, increased SOD activity and restored proliferation of granulosa cells [168]. Curcumin was also able to stimulate proliferation and counteract the apoptotic effect of whole-body ionizing radiation on mouse follicular cells [169].

Acting as an antioxidant, curcumin was able to inhibit the lysophosphatidic acid-dependent NF-κB activity and cell proliferation of OC cells, SKOV3 line [170]. Importantly, when assayed in an orthotropic murine OC model, curcumin significantly blocked the activation of NF-κB transcription factor, leading to growth suppression and inhibition of angiogenesis [171]. In the OC model, daily dietary curcumin intake reduced the frequency of spontaneous ovarian tumors in up to 44% respect to the unfed control during a 12-months period [172]. Curcumin was able to repress the expression of the cancer stem cell marker ALDH1A1 in OC cells leading to enhanced sensitivity to cisplatin, and also showed capacity to impair adhesion and invasion of OC spheroids to the extracellular matrix and to mesothelial monolayers, thus showing potential as an anti-metastatic agent [173]. Its long-term safety coupled to minimal side-effects suggest that curcumin might be applied to OC treatment, particularly in platinum-resistant recurrent or multidrug resistant OC patients [174].

## COMMON PATHWAYS OF ROS-MEDIATED TUMORIGENESIS IN FEMALE CANCERS

Although physiological levels of reactive species function as important signaling of certain subcellular events such as gene expression [175], protein synthesis [176], and enzymatic activity [177], elevated levels of reactive species could initiate multiple toxic oxidative reactions including initiation by lipid peroxidation, among others that are known to play a role in carcinogenesis [178–180]. In addition, elevated levels of reactive species could alter and damage many intracellular molecules, including nucleic acids, proteins, lipids, and polysaccharides [181], thus initiating a series of pathological processes and diseases.

**Figure 1:**
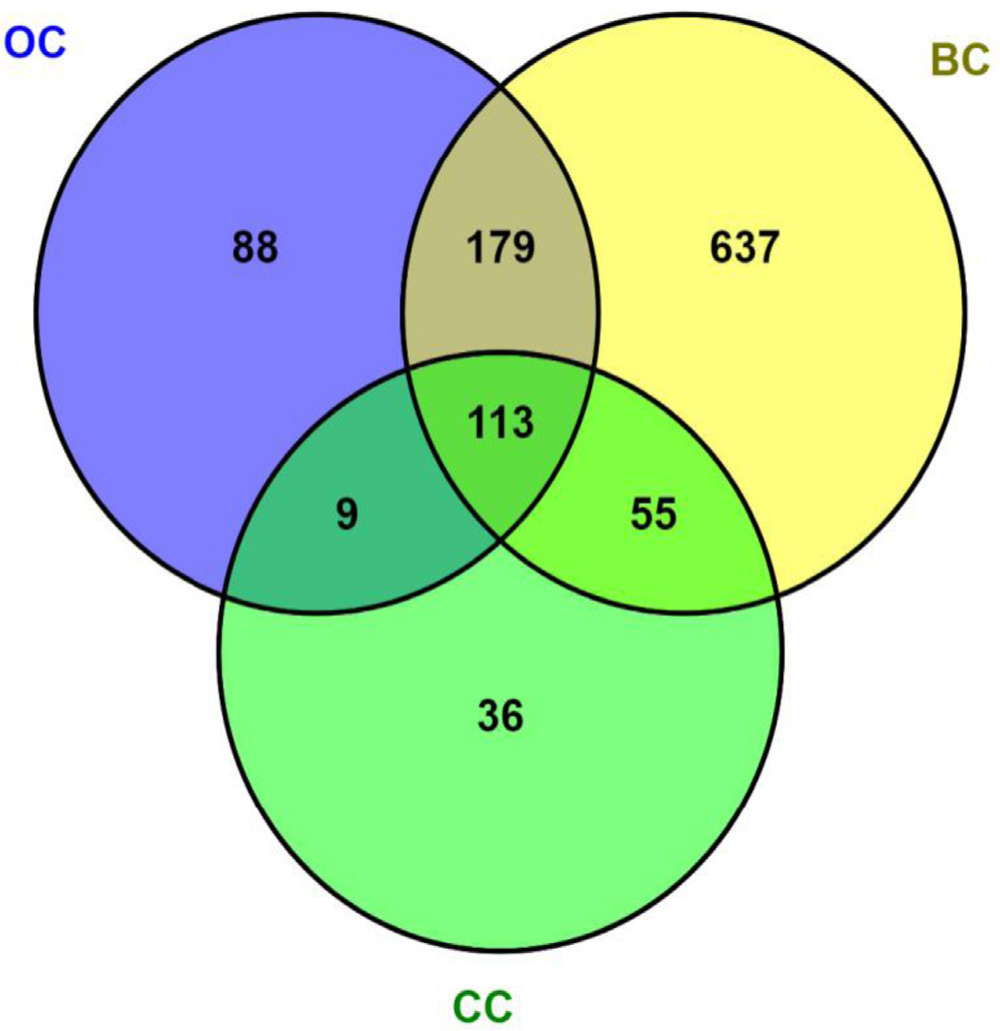
Systematic literature analysis of genes associated to oxidative stress in female cancers PubMed was used via the text-mining tool SciMiner (http://hurlab.med.und.edu/SciMiner/, accessed August 1st, 2017) with the keywords “breast cancer” and “ROS”, “cervical cancer” and “ROS”, and “ovarian cancer” and “ROS” in separate queries. Using the default settings of SciMiner, the output resulted in 984, 213 and 289 breast cancer (BC), cervical cancer (CC) and ovarian cancer (OC)-associated genes, respectively. The Venn diagram was obtained with VENNY 2.1 (Oliveros JC, 2007–2015. *Venny. An interactive tool for comparing lists with Venn's diagrams*. http://bioinfogp.cnb.csic.es/tools/venny/index.html). The resultant list of 113 genes overlapping the 3 queries was subjected to the gene ontology (GO) analysis described in Table 1.

Therefore, aiming to cover this wide spectrum of molecular alterations inflicted to the cellular machinery by OS, we performed a systematic text-mining approach that is summarized in Figure 1. By using the open-access tool SciMiner [182], a set of 113 genes associated to breast (BC), cervical (CC) and ovarian (OC) cancers was identified. When disease pairs were considered, the highest overlap was observed between BC and OC (292 genes) followed by BC compared with CC (168 genes). The minimal coincidence occurred between CC and OC (122 genes). As we recognize that the sizes of these gene-sets might reflect historic and research-priority bias, but not truly biological divergences, we focused in the coincident 113 gene subset to perform a gene ontology (GO) analysis. GO is a biological knowledge tool describing gene function as a compendium of terms/concepts aimed to accurately classify gene products in 3 domains: i) the biological process(es) in which they participate; ii) the molecular function(s) they display and iii) the cellular compartment/component(s) in which they reside or act. GO terms are described in a precise, unambiguous vocabulary and a species-independent manner [183]. The results of GO analysis of the 113 genes coincident among BC, CC and OC are shown in Table 1.

**Table 1:** Gene ontology profile of genes related to oxidative stress in female cancers^*^

Go term	Number of genes	Q
Regulation of cell death	72	1.75E–43
Nuclear part	66	3.16E–14
Regulation of transcription, DNA-templated	57	3.10E–11
Cell surface receptor signaling pathway	50	3.09E–15
Regulation of phosphorylation	50	7.98E–23
Response to oxidative stress	42	7.16E–39
Regulation of cell cycle	41	7.73E–20
Mitochondrion	40	8.11E–13
Response to DNA damage stimulus	32	7.24E–17
Response to metal ion	32	3.88E–27
Regulation of kinase activity	30	1.24E–13
Response to hormone	28	7.88E–13
Regulation of MAPK cascade	28	5.61E–14
Response to hypoxia	22	4.30E–15
Chromatin organization	13	1.26E–03
DNA repair	10	4.15E–03
Cellular oxidant detoxification	10	1.45E–09
Antioxidant activity	10	3.36E–09

^*^The list of 113 coincident genes implicated in BC, CC and OC (see Figure 1) were analysed with the GO tool VLAD. Note that some degree of gene overlap might occur among the indicated GO terms. “Q” indicates the false discovery rate, i.e. the multiple test adjusted significance value.

Cancer cells usually exhibit increased levels of reactive species [184], which are found to facilitate cancer growth through sustained proliferation, apoptosis resistance, death evasion, angiogenesis, invasiveness, and metastasis [185–188]. Therefore, eliminating elevated oxidative stress is considered as an important strategy for cancer prevention [168]. DNA damage is a complex process involving multiple steps as DNA repair, cell survival and cell death pathways [189–192] that includes single strand breaks, double-strand break (DSB), base damage, bulky adducts, intra/inter strand cross links, and breakdown of replication fork lesions. One of the key proteins in the base excision repair pathway is the Poly Adenosine Diphosphate Ribose Polymerase-1 (PARP-1) [193–196] that is a 116-kDa nuclear protein that appears to be involved in DNA repair in response to environmental stress [195, 196]. It is an important single strand break binding protein that is cleaved in response to DNA damage in cells undergoing apoptosis. Thus, PARP serves as a marker of cells undergoing apoptosis. We determined the effect of curcumin on DNA damage and showed that curcumin (30 μM) stimulated cleaved PARP-1 protein expression in control MCF-10F, estrogen, Alpha3 and Tumor2 suggesting that curcumin induced apoptosis in breast cancer cells [66]. Reactive species could also cause nicks in DNA, as well as malfunctions in the DNA repair mechanism. DNA oxidation induced by these reactive species generates 8- hydroxy-2′-deoxyguanosine, a product that is able to generate mutations in DNA and enhances carcinogenesis [66, 197].

Accumulation of phosphorylated histone H2AX, also called γ-H2AX, is a marker for DSB [190–192]. The H2AX, member of the family H2A is a subunit of the nucleosome that phosphorylate at the serine 139 in the C-tail serine-glutamine-glutamate motif that is one of the earliest responses of mammalian cells to ionizing radiation-inducing DNA-DSB [190–192]. H2AX forms discrete foci at the sites of DSBs and facilitates the remodeling complexes to the sites of DNA damage influencing both the efficiency and fidelity of DSB repair.

It is known that curcumin interferes with the transcription activation induced by transcription factors such as nuclear factor-κB (NF-κB), resulting in the negative regulation of various cell cycle control genes and oncogenes [198, 199]. The nuclear NF-κB complex containing p65 (Rel A) and p50 consists of proteins that act as a multifunctional nuclear transcription factor [200] that regulates the expression of multiple genes promoting carcinogenesis. Activators of nuclear NF-κB include various cellular stressors such as environmental carcinogens, tumor promoters, ROS, apoptosis inducers, among others. Activation of NF-κB has been implicated in resistance of cancer cells to radiotherapy and chemotherapy [198]. It has been reported that curcumin may inhibit NF-κB and may regulate DNA binding in pancreatic cancer cells, inducing apoptosis [152, 201–203]. Our results indicated that curcumin decreased NF-κB protein expression of MCF-10F, Alpha3, Alpha5, and Tumor2 cell lines in comparison to their control counterparts [66].

## CONCLUSIONS AND REMARKS

It can be concluded that epidemiological, experimental and clinical studies have found a role for free radicals that are enhanced in diseases as cancer. Oxidative stress is closely connected with the initiation and progression of cancer. The body of evidence indicates that ROS can induce, promote and modulate carcinogenesis Anti-oxidation system as enzymatic and non-enzymatic antioxidants defends against OS, including oxidation and reduction of molecules that lead to free radical production. We reviewed the recent progress toward the potential role of ROS and associated OS in carcinogenesis since they are involved in the development and progression of several human cancers like cervical, breast and ovary. Future avenues: Therefore, research focusing on cancer samples are urgent for a better understanding the role of OS-induced cancer initiation and progression, as well as its potential as an OS related protein for cancer antioxidant prevention.
